# Outcomes of hospitalized hematologic oncology patients receiving rapid response system activation for acute deterioration

**DOI:** 10.1186/s13054-019-2568-5

**Published:** 2019-08-27

**Authors:** Benjamin Gershkovich, Shannon M. Fernando, Brent Herritt, Lana A. Castellucci, Bram Rochwerg, Laveena Munshi, Sangeeta Mehta, Andrew J. E. Seely, Daniel I. McIsaac, Alexandre Tran, Peter M. Reardon, Peter Tanuseputro, Kwadwo Kyeremanteng

**Affiliations:** 1 Division of Critical Care, Department of Medicine, University of Ottawa, Ottawa, ON Canada; 2 Department of Emergency Medicine, University of Ottawa, Ottawa, ON Canada; 3 School of Epidemiology and Public Health, University of Ottawa, Ottawa, ON Canada; 4 Clinical Epidemiology Program, Ottawa Hospital Research Institute, Ottawa, ON Canada; 5 Division of Hematology, Department of Medicine, University of Ottawa, Ottawa, ON Canada; 6 Department of Medicine, Division of Critical Care, McMaster University, Hamilton, ON Canada; 7 Department of Health Research Methods, Evidence, and Impact, McMaster University, Hamilton, ON Canada; 8 Department of Medicine, Sinai Health System, and Interdepartmental Division of Critical Care Medicine, University of Toronto, Toronto, ON Canada; 9 Department of Surgery, University of Ottawa, Ottawa, ON Canada; 10 Department of Anesthesiology and Pain Medicine, University of Ottawa, Ottawa, ON Canada; 11 Division of Palliative Care, Department of Medicine, University of Ottawa, Ottawa, ON Canada; 12 Institut du Savoir Montfort, Ottawa, ON Canada; 13 The Ottawa Hospital, 501 Smyth Rd, Ottawa, ON K1H 8L6 Canada

**Keywords:** Critical care, Malignancy, Mortality, Chemotherapy, Hematology, Sepsis

## Abstract

**Background:**

Patients with hematologic malignancies who are admitted to hospital are at increased risk of deterioration and death. Rapid response systems (RRSs) respond to hospitalized patients who clinically deteriorate. We sought to describe the characteristics and outcomes of hematologic oncology inpatients requiring rapid response system (RRS) activation, and to determine the prognostic accuracy of the SIRS and qSOFA criteria for in-hospital mortality of hematologic oncology patients with suspected infection.

**Methods:**

We used registry data from two hospitals within The Ottawa Hospital network, between 2012 and 2016. Consecutive hematologic oncology inpatients who experienced activation of the RRS were included in the study. Data was gathered at the time of RRS activation and assessment. The primary outcome was in-hospital mortality. Logistical regression was used to evaluate for predictors of in-hospital mortality.

**Results:**

We included 401 patients during the study period. In-hospital mortality for all included patients was 41.9% (168 patients), and 145 patients (45%) were admitted to ICU following RRS activation. Among patients with suspected infection at the time of RRS activation, Systemic Inflammatory Response Syndrome (SIRS) criteria had a sensitivity of 86.9% (95% CI 80.9–91.6) and a specificity of 38.2% (95% CI 31.9–44.8) for predicting in-hospital mortality, while Quick Sequential Organ Failure Assessment (qSOFA) criteria had a sensitivity of 61.9% (95% CI 54.1–69.3) and a specificity of 91.4% (95% CI 87.1–94.7). Factors associated with increased in-hospital mortality included transfer to ICU after RRS activation (adjusted odds ratio [OR] 3.56, 95% CI 2.12–5.97) and a higher number of RRS activations (OR 2.45, 95% CI 1.63–3.69). Factors associated with improved survival included active malignancy treatment at the time of RRS activation (OR 0.54, 95% CI 0.34–0.86) and longer hospital length of stay (OR 0.78, 95% CI 0.70–0.87).

**Conclusions:**

Hematologic oncology inpatients requiring RRS activation have high rates of subsequent ICU admission and mortality. ICU admission and higher number of RRS activations are associated with increased risk of death, while active cancer treatment and longer hospital stay are associated with lower risk of mortality. Clinicians should consider these factors in risk-stratifying these patients during RRS assessment.

**Electronic supplementary material:**

The online version of this article (10.1186/s13054-019-2568-5) contains supplementary material, which is available to authorized users.

## Background

Hematologic oncology patients admitted to hospital are at high risk of short-term deterioration, due to the nature of their underlying disease processes as well as the chemotherapeutic regimens that they receive [1]. Common complications that lead to clinical deterioration, ICU admission, and/or death include infection, organ-specific toxicities related to chemotherapy, and sequelae of the malignancies themselves, such as tumor-lysis syndrome, leukostasis, or bleeding [2–5].

In an attempt to mitigate the adverse outcomes associated with in-hospital deterioration, rapid response systems (RRSs) have been widely implemented internationally [6]. These teams consist of multi-disciplinary groups of health care providers who respond to in-hospital emergencies and provide acute management, transfer patients to higher level of care settings, or assist with goals-of-care discussions and palliation [7, 8]. The use of RRSs has been associated with lower rates of in-hospital mortality and cardiac arrest, lower rates of readmission to ICU following ICU discharge, and earlier initiation of goals-of-care discussions and facilitation of end-of-life care [9–16].

With respect to the potential causes of deterioration among hematologic oncology inpatients, community-acquired and nosocomial infections are common and account for significant morbidity and mortality [17–19]. Risk stratification of patients with suspected infection is extremely valuable in early initiation of treatment, escalation of care, and evaluation of goals of care. Several tools, including the SIRS criteria [20] and qSOFA score [21], may be used to risk-stratify patients with suspected infection. The use of these risk-stratification tools among inpatients with suspected infection who require RRS activation has been described previously [22, 23]; however, there is a paucity of data with respect to the use of these tools in this specific population.

The outcomes of hematologic oncology patients admitted to the ICU have been described previously [24–28]. A recent retrospective cohort study of 1097 ICU patients with hematologic malignancies reported 28-day, 3-month, and 1-year survival rates of 56%, 48%, and 38% respectively [29]. However, very few previous studies have examined whether or not these outcome data can be extrapolated upstream of ICU admission to patients who deteriorate in-hospital. Taheri et al. [30] recently performed a retrospective analysis of 126 hematologic oncology patients who were assessed by a critical care outreach team and found that a diagnosis of sepsis was associated with an increased risk of mortality for those admitted to ICU. Additionally, several studies have examined early versus late ICU admission among hematologic oncology patients and have consistently found correlations between delayed ICU admission and increased mortality [31–33]. RRS implementation may result in earlier ICU admission for appropriately selected hematologic oncology and hematopoietic cell transplant (HCT) patients, leading to lower mortality rates [34–36].

We sought to examine the outcomes of hospitalized hematologic oncology patients who experience RRS activation for deterioration and to determine the factors that are independently associated with in-hospital mortality. Finally, given the high prevalence of infection and infectious complications among this population, we aimed to examine the prognostic accuracy of the SIRS criteria and the qSOFA criteria for prediction of in-hospital mortality among hematologic oncology patients with suspected infection.

## Methods

### Study design, setting, and subjects

Ethics approval was obtained from The Ottawa Health Science Network Research Ethics Board. This study was performed at two tertiary care hospitals within The Ottawa Hospital network. Each of the two hospitals has its own 28-bed tertiary medical-surgical ICU, with 2500 combined ICU admissions per year. The Ottawa Hospital Network is a regional HCT and Oncology centre.

All data was obtained from the Ottawa Hospital Data Warehouse, a clinical and health administrative database that has been used in previous research [22, 37]. Quality of the gathered data is routinely confirmed through the use of regular quality-assurance checks.

We retrospectively evaluated patients with hematologic malignancies or HCT who were over the age of 18 and had undergone RRS activation at one of the participating hospitals between 2012 and 2016. Included patients were admitted under either the malignant hematology service or the HCT service, both of which admit patients with hematologic malignancies. We did not use specific exclusion criteria; however, patients were excluded if they did not meet the above inclusion criteria. “HCT patients” included all patients who had received at least one allogeneic or autologous HCT at any time prior to RRS activation. Patients were considered to be receiving active treatment if they were in the middle of a course of either chemotherapy or radiation therapy, regardless of cycle timing. For example, a patient who had completed a cycle of chemotherapy 2 weeks prior to RRS activation would qualify for inclusion if one or more cycles of chemotherapy remained within their course of treatment. Active treatment also included pre-HCT induction chemotherapy. Criteria for RRS activation at our institution have been published previously [38] and include respiratory distress, arrhythmia, and hypotension (Additional file 1: Table S1). However, all healthcare providers are encouraged to activate the RRS for any serious patient-related concerns. The RRS at our institution is comprised of a critical care nurse and respiratory therapist, each with several years of ICU experience, as well as 1–2 MDs. The RRS is capable of providing vasoactive and inotropic medications, respiratory support including high-flow nasal cannula and non-invasive positive pressure ventilation, and intubation. All prescribing of medications is performed by MDs. Background specialty and level of training is variable among MDs on the RRS, but specialties include internal medicine, surgery, anesthesia, and emergency medicine.

### Data collection

Baseline patient data, including demographics, comorbidities, and Elixhauser comorbidity scores, as well as ED visits, hospital admissions, and ICU admissions in the past year had been previously abstracted at the time of patient admission and stored in the Ottawa Hospital Data Warehouse. Data about specific patient diagnoses, HCT, active therapy, neutropenia, and mortality were abstracted from the electronic medical record by a single reviewer (BG). Data related to RRS activation was abstracted by trained RRS nurses at the time of RRS assessment, stored in the Data Warehouse, and used to calculate SIRS and qSOFA scores. These data included most recent vital signs and laboratory values at the time of activation, time of RRS activation, reason for activation, admitting service, and ICU admission following RRS assessment. Data on latency time from onset of concerning symptoms/signs (e.g., hypotension, altered mental status) to initial RRS activation are also estimated at the time of RRS assessment, as part of a quality improvement initiative.

The primary outcome was in-hospital mortality. Secondary outcomes included ICU admission after RRS activation, ICU length of stay, total hospital length of stay, and percentage of survivors discharged home who were originally from home prior to admission. A secondary analysis was performed to determine the prognostic accuracy of the SIRS (Additional file 2: Table S2) and qSOFA criteria for in-hospital mortality of hematologic oncology patients with suspected infection. Suspected infection was defined as concomitant administration of oral or parenteral antibiotics and sampling of body fluid cultures (blood, urine, cerebrospinal fluid, peritoneal, etc.). If cultures were drawn first, antibiotics had to be administered within 72 h, whereas if the antibiotic was administered first, cultures had to be drawn within 24 h. This definition is in keeping with the Sepsis-3 definition of suspected infection [21].

### Statistical analysis

All of the statistical analyses were performed with commercially available statistical packages (R, Version 3.3.3, and IBM SPSS, Version 24.0). Data are presented as mean values (with standard deviation, SD), or as medians (with interquartile range, IQR), where indicated. Descriptive statistics were utilized for between-group comparisons using Student’s *t* test (for normally distributed values), the Mann-Whitney test (for non-normally distributed values), and *χ*^2^ (for categorical values). To evaluate predictors of mortality, a logistic regression model was created based on a priori selection of clinically important variables including patient demographics, medical comorbidities, and RRS activation characteristics. We adhered to an event per variable ratio > 10 [39] and intentionally avoided selection methods based on univariate testing in order to minimize potential overfitting [40].

## Results

During the study period, the RRS was activated for 6132 patients. Of these, 401 patients met our inclusion criteria and were included in the analysis (Additional file 4: Figure S1). One hundred and forty-five patients (36.2%) had received HCT prior to RRS activation. The baseline characteristics of the included patients are summarized in Table 1. Leukemia was the most prevalent hematologic malignancy in our population (39.2%), followed by lymphoma (32.7%) and multiple myeloma (14.7%). Three hundred and forty-one patients (85.0%) were admitted from home, and 300 patients (74.8%) were classified as having “full” goals of care, which allowed for cardiopulmonary resuscitation, intubation, and transfer to ICU.

**Table 1:** Baseline characteristics of hematologic oncology and subgroup of HCT patients

Variables	All hematologic oncology patients (*n* = 401)	HCT patients (*n* = 145)
Age, years, mean (SD)	61.6 (15.3)	53.1 (14.3)
Male, *n* (%)	254 (63.3)	88 (60.7)
Hematologic diagnosis		
Leukemia	157 (39.2)	82 (56.6)
Lymphoma	131 (32.7)	35 (24.1)
Multiple myeloma	60 (14.7)	10 (6.9)
Other	53 (13.2)	18 (12.4)
Admission source, *n* (%)		
Home	341 (85.0)	123 (84.8)
Acute care facility transfer	21 (5.2)	11 (7.6)
Long-term care facility transfer	31 (7.7)	9 (6.2)
Other	8 (2.0)	2 (1.4)
Comorbidities, *n* (%)		
Congestive heart failure	43 (10.7)	6 (4.1)
Arrhythmia	66 (16.5)	28 (19.3)
Valvular disease	5 (1.2)	2 (1.4)
Peripheral vascular disease	2 (0.5)	1 (0.7)
Hypertension	80 (20.0)	23 (15.9)
Chronic obstructive pulmonary disease	30 (7.5)	8 (5.5)
Diabetes mellitus	129 (32.2)	45 (31.0)
Renal failure	26 (6.5)	4 (2.8)
Liver disease	17 (4.2)	10 (6.9)
Metastatic cancer	15 (3.7)	6 (4.1)
Elixhauser comorbidity score, mean (SD)	9.6 (8.2)	7.7 (6.6)
Emergency department visits in past year, median (IQR)	1 (0–2)	0 (0–1)
Hospital admissions in the past year, median (IQR)	1 (0–2)	1 (0–3)
ICU admissions in the past year, median (IQR)	0 (0–0)	0 (0–0)
Limits of care, *n* (%)		
Full care	300 (74.8)	126 (86.9)
ICU-level care, no CPR	22 (5.5)	4 (2.8)
Do not resuscitate	56 (14.0)	6 (4.1)
Other/unknown	23 (5.7)	9 (6.2)

*Abbreviations*: *RRS* rapid response system, *SD* standard deviation, *IQR* interquartile range, *ICU* intensive care unit, *CPR* cardiopulmonary resuscitation

The characteristics of the included RRS calls are detailed in Table 2. Two hundred and forty-six patients across the entire cohort (61.3%) and 70 patients (48.3%) in the HCT group were actively receiving treatment at the time of RRS activation. One hundred and fifty-four patients (38.4%) were neutropenic at the time of RRS activation, and the most common reason for RRS activation was respiratory distress (124 patients, 30.9%).

**Table 2:** Characteristics of initial RRS call in hematologic oncology and HCT patients

Variables	All hematologic oncology patients (*n* = 401)	HCT patients (*n* = 145)
Rapid response system activations during admission, median (IQR)	1 (1–1)	1 (1–1)
Time of initial RRS activation		
Activations during working hours (0800–1659)	190 (47.4)	73 (50.3)
Most recent vital signs		
Systolic blood pressure, mmHg, mean (SD)	118 (27)	120 (25)
Diastolic blood pressure, mmHg, mean (SD)	70 (15)	71 (15)
Heart rate, beats/min, mean (SD)	108 (29)	112 (28)
Temperature, degrees Celsius, mean (SD)	37.0 (0.9)	37.1 (0.8)
Oxygen saturation, %, median (IQR)	95 (93–97)	95 (93–97)
Most recent blood work		
White blood cell count, ×10^9^/L, median (IQR)	4.7 (1.0–12.2)	1.9 (0.1–8.3)
Hemoglobin, g/L, mean (SD)	95 (19)	91 (17)
Platelets, ×10^9^/L, mean (SD)	87 (102)	47 (65)
Potassium, mmol/L, mean (SD)	4.0 (0.7)	3.9 (0.7)
Creatinine, μmol/L, median (IQR)	83 (60–138)	76 (57–109)
Urea, mmol/L, median (IQR)	7.4 (4.7–12.3)	6.7 (4.3–10.9)
Lactate, mmol/L, median (IQR)	2.0 (1.5–3.1)	1.9 (1.5–3.2)
Albumin, g/L, mean (SD)	24 (6)	24.2 (6.0)
INR, median (IQR)	1.3 (1.1–1.4)	1.3 (1.1–1.4)
Active treatment	246 (61.3)	70 (48.3)
Neutropenia	154 (38.4)	70 (48.3)
Time to first RRS activation from onset of concerning symptoms/signs		
< 1 h	246 (61.3)	81 (55.9)

*Abbreviations*: *RRS* rapid response system, *SD* standard deviation, *IQR* interquartile range, *ICU* intensive care unit

**Fig. 1:**
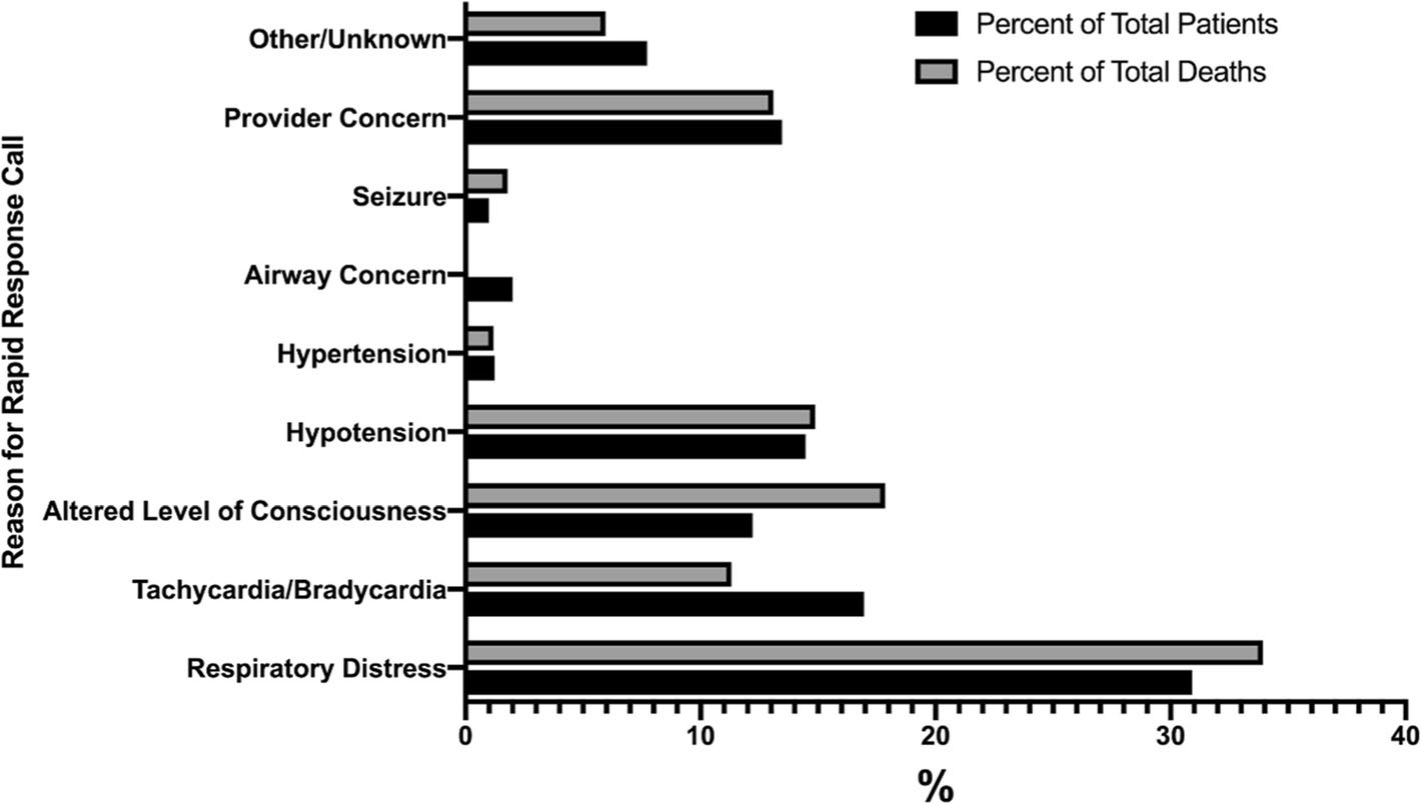
Reasons for rapid response system activation in hematologic oncology patients

A total of 145 patients (45.0%) were transferred to the ICU after their RRS call, and the in-hospital mortality rate for all patients was 41.9% (Table 3). Within the HCT subgroup, 55 patients (41.7%) were transferred to the ICU and 53 patients (36.6%) died in hospital. There were no significant differences in mortality or need for ICU admission between the HCT and non-HCT groups (*p* > 0.05 for both comparisons). The most common reason for RRS activation was respiratory distress, followed by dysrhythmias (Fig. 1). As a reason for activation, respiratory distress also accounted for the highest proportion of RRS calls that resulted in mortality (Fig. 1).

**Table 3:** Outcomes of hematologic oncology and HCT patients requiring rapid response system activation

Outcomes	All hematologic oncology patients (*n* = 401)	HCT patients (*n* = 145)
Mortality, *n* (%)	168 (41.9)	53 (36.6)
Admit to ICU, *n* (%)^a^	145 (45.0)	55 (41.7)
ICU length of stay, days, median (IQR)	5 (1–9)	6 (2–9)
Hospital length of stay, days, median (IQR)	19 (8.5–37)	26.5 (16–47)
Survivors discharged home, *n* (%)^b^	154 (76.2)	61 (77.2)

^a^Analysis only includes patients with goals of care allowing for ICU admission (hematology oncology: *n* = 322; HCT: *n* = 13))

^b^Analysis only includes survivors initially from home (hematology oncology: *n* = 202; HCT: *n* = 79)

*Abbreviations*: *RRS* rapid response system, *ICU* intensive care unit, *IQR* interquartile range, *CI* confidence interval

Among included patients, 318 (79.3%) met criteria for suspected infection, and the most common suspected source of infection was pulmonary (Table 4). The SIRS criteria had a sensitivity of 86.9% (95% CI 80.9–91.6) and a specificity of 38.2% (95% CI 31.9–44.8) for predicting in-hospital mortality. For the same population, the qSOFA criteria had a sensitivity of 61.9% (95% CI 54.1–69.3) and a specificity of 91.4% (95% CI 87.1–94.7) for prediction of in-hospital mortality.

**Table 4:** Prognostic accuracy of sepsis criteria in hematology oncology and HCT patients with suspected infection

Variables	All hematology oncology patients (*n* = 318)	HCT patients (*n* = 122)
Suspected source of infection, *n* (%)		
Pulmonary	151 (47.5)	55 (45.1)
Gastrointestinal	89 (28.0)	37 (30.3)
Urinary tract	35 (11.0)	13 (10.7)
Skin/soft tissue	10 (3.1)	4 (3.3)
Central nervous system	6 (1.9)	3 (2.5)
Other/unknown	27 (8.5)	10 (8.2)
Disease severity, median (IQR)		
Total SOFA score	3 (1–5)	3 (2–5)
Respiratory SOFA score	1 (0–3)	1 (0–3)
Coagulation SOFA score	1 (0–1)	1 (1–2)
Liver SOFA score	0 (0–0)	0 (0–0)
Cardiovascular SOFA score	1 (1–3)	1 (1–3)
Central nervous system SOFA score	0 (0–1)	0 (0–1)
Renal SOFA score	1 (0–2)	1 (0–2)
SIRS criteria		
Sensitivity, % (95% CI)	86.9 (80.9–91.6)	92.5 (81.8–97.9)
Specificity, % (95% CI)	38.2 (31.9–44.8)	25.0 (16.6–35.1)
Positive predictive value, % (95% CI)	50.3 (47.4–53.3)	41.5 (38.2–44.5)
Negative predictive value, % (95% CI)	80.2 (72.6–86.1)	85.2 (67.8–94.0)
Positive likelihood ratio (95% CI)	1.41 (1.25–1.58)	1.23 (1.07–1.42)
Negative likelihood ratio (95% CI)	0.34 (0.22–0.52)	0.30 (0.11–0.83)
qSOFA criteria		
Sensitivity, % (95% CI)	61.9 (54.1–69.3)	64.2 (49.8–76.9)
Specificity, % (95% CI)	91.4 (87.1–94.7)	88.0 (79.6–93.9)
Positive predictive value, % (95% CI)	83.9 (77.1–88.9)	75.6 (63.2–84.8)
Negative predictive value, % (95% CI)	76.9 (73.2–80.2)	81.0 (74.7–86.0)
Positive likelihood ratio (95% CI)	7.21 (4.67–11.15)	5.37 (2.97–9.68)
Negative likelihood ratio (95% CI)	0.42 (0.34–0.51)	0.41 (0.28–0.59)

*Abbreviations*: *CI* confidence interval, *IQR* interquartile range, *SOFA* Sequential Organ Failure Assessment

The results of the multivariate logistic regression analysis are presented in Additional file 3: Table S3. Factors that were independently associated with in-hospital mortality included ICU admission (OR 3.56, 95% CI 2.12–5.97), more than one RRS activation during admission (OR 2.45, 95% CI 1.63–3.69), goals of care that did not allow for cardiopulmonary resuscitation (OR 3.57, 95% CI 1.53–8.32), and goals of care that did not allow for ICU admission (OR 2.83, 95% CI 1.42–5.61). Factors that were associated with lower likelihood of in-hospital mortality included active malignancy treatment (OR 0.54, 95% CI 0.34–0.86) and longer length of hospital stay (OR 0.78, 95% CI 0.70–0.87).

## Discussion

The outcomes of hematologic oncology patients admitted to the ICU have been described previously [24–28]. However, very few previous studies have examined whether or not these outcome data can be extrapolated upstream of ICU admission to patients who deteriorate in-hospital. We have shown that hematologic oncology inpatients who require RRS activation experience high rates of ICU admission and mortality. Additionally, we have described the sensitivity and specificity of both the qSOFA and SIRS criteria with respect to prediction of in-hospital mortality for these patients. Finally, we have delineated several factors associated with increased risk of mortality in this population, which include ICU admission, more than one RRS activation, and goals of care that allow for fewer aggressive interventions. Few studies have previously examined these factors as they relate to the hematologic oncology population during RRS calls.

We found that hematologic oncology inpatients who experience RRS activation had an in-hospital mortality rate of 41.9%, which is higher than mortality among other previously studied RRS populations, including the elderly [37], general oncology patients [41], and surgical patients [42] (36.2%, 33%, and 28.4%, respectively). In our database, in-hospital mortality among non-hematologic oncology RRS patients was 29.9% [43], suggesting markedly higher mortality among the hematologic oncology population, as expected. This suggests that the hematologic oncology population is uniquely vulnerable, which may be due to the risk of infection and treatment-related complications in these patients, as described earlier.

Previous studies have examined mortality of hematologic oncology patients admitted to the ICU [26, 44]. Most recently, de Vries et al. [29] described a 28-day survival rate of 56% among hematologic oncology patients admitted to the ICU. Our data suggests that mortality after RRS activation is similar to mortality rates after ICU admission. This information may be of use to RRS clinicians, as prognostication of outcomes among these patients may begin upstream of ICU admission, at the time of assessment following deterioration. The high risk of mortality in this population emphasizes the need for health care providers to appropriately assess these patients using prognostic tools prior to making the decision to transfer to the ICU.

We describe several unique factors associated with in-hospital mortality in the hematologic oncology population after RRS activation, which add novel insights to the growing body of literature describing the outcomes and prognostic variables among this patient population [30]. Goals-of-care designations that allow for fewer aggressive interventions were associated with higher risk of mortality in our study. This is to be expected, given that these patients receive fewer life-sustaining therapies and tend to be palliated sooner than those who are admitted to the ICU and undergo mechanical ventilation and cardiopulmonary resuscitation [45].

ICU admission itself was also associated with increased mortality in our study. This is consistent with previous data comparing outcomes of ICU versus non-ICU hematologic oncology patients. Jacobson et al. [46] found that in a sample of 656 hematologic oncology patients, in-hospital mortality was 33% for patients admitted to the ICU, compared to 8% for those who were not. Long-term survival rates were also better in non-ICU patients in this study. This highlights the importance of appropriate triaging and decision-making for hematologic oncology ward patients who acutely deteriorate, as ICU admission should ideally only occur for those patients who can predictably benefit from it.

Our data show that compared with a single RRS activation, more than one RRS call for a given patient during a single admission is associated with increased mortality during the same admission. This is consistent with previous data demonstrating a relationship between increased number of RRS calls and higher risk of in-hospital mortality, after adjusting for patient factors [47, 48]. While there are few significant differences between patients with single versus multiple RRS calls [47], a higher number of RRS activations may reflect instability or frailty. Additionally, this may signify the need to use a lower ICU admission threshold for this vulnerable population, such that prolonged ward deterioration and subsequent negative outcomes may be avoided.

Azoulay et al. [27] previously demonstrated that hematologic oncology patients whose malignancies were in complete or partial remission were less likely to die in hospital after ICU admission. The same association was described for patients with solid tumors admitted to the ICU [49]. Although our data did not capture remission status of the included patients, we found that active malignancy treatment in the form of chemotherapy and/or radiation is associated with a lower risk of in-hospital mortality among hematologic oncology patients. Following ICU admission, ongoing chemotherapy has been associated with longer length of hospital and ICU stays, but mortality in these patients is similar to those who do not receive chemotherapy [50]. In our study, the association between active treatment and reduced mortality may be influenced by residual confounding, as those who receive active treatment may be healthier and may therefore experience more favorable outcomes following ICU admission. Nonetheless, these data may help RRS clinicians with decision making, as it appears that those patients receiving active treatment at the time of deterioration may benefit from ICU admission and aggressive intervention.

As mentioned, the association between early versus late ICU admission and improved survival in hematologic oncology patients has been established previously. Though there is variability among the studies that have examined this association, rates of in-hospital mortality for early ICU-admitted hematologic oncology patients ranges from 20 to 65% [31, 32, 51]. Our reported mortality rate lies centrally within this range, and further studies are required to elucidate whether or not RRS activation can facilitate earlier ICU admission in this population, and therefore lead to improved outcomes.

One potential explanation for the benefits of early ICU admission is that deterioration and death in this population can occur in a dramatically rapid fashion as a result of the previously mentioned high-risk features in these patients such as immunosuppression [31, 33, 52]. This concept may also partly explain the association between longer hospital stay and lower risk of mortality in our study, as patients who experienced rapid deterioration and death, and therefore shorter length of hospital stay, may have represented a large proportion of those who died in hospital.

Infectious complications occur at high rates in the hematologic oncology population and are responsible for significant morbidity and mortality [17–19]. Our data confirm that infection is a major component of in-hospital deterioration of hematologic oncology patients, as 79.3% of the patients included in our study met criteria for suspected infection. The SIRS and qSOFA tools have been applied to other in-patient populations with suspected infection for risk stratification [22, 23, 53]; however, ours is the first study to examine their use in hematologic oncology patients.

We found that the SIRS criteria were more sensitive while the qSOFA criteria were more specific with respect to prognostic accuracy of in-hospital mortality for hematologic oncology patients with suspected infection. This is in keeping with recent data suggesting similar associations in patients with suspected infection who undergo RRS activation [22], as well as the observation that higher mortality rates are seen among ICU patients when Sepsis-3 criteria are applied, as compared with Sepsis-2 criteria [54]. It is important to note that in the general population, SIRS and qSOFA patients are not entirely similar, and each prognostic score must be evaluated in context. There appears to be a role for both the SIRS and qSOFA criteria in this population, and both scoring systems may be used in a complementary fashion. The value of the SIRS criteria in these patients lies in screening. For those who do meet SIRS criteria, however, the qSOFA criteria should then be used for risk stratification upstream of ICU admission, given their high specificity with respect to in-hospital death. This is especially important given the previously mentioned observation that appropriately selected hematologic oncology patients who are admitted to ICU early may experience lower mortality rates.

This study has several strengths. We used a previously validated database that includes data from two hospitals within a single hospital network. Additionally, this is one of few studies to examine outcomes of hematologic oncology patients in the context of RRS activation. The study’s limitations include lack of access to data that would identify the impact of RRS activation on goals-of-care designation. It is therefore unclear if RRS activation significantly influences medical directive changes and subsequently rates of mortality in this population. Additionally, the criteria used to trigger RRS calls at our institution may lead to delays in the detection of specific clinical entities such as sepsis, as compared with other triggering criteria such as the qSOFA or National Early Warning (NEWS) scores. Finally, the ability to include HCT in the multivariate analysis was hindered by sample size. Though there was no difference in mortality between HCT and non-HCT patients in our study, historically allogenic HCT in particular has been described as a prognostic factor for poor outcomes in hematologic oncology patients admitted to ICU [55]. More data is required to adequately describe the potential prognostic significance of HCT in this population. With respect to other prognostic factors, further research should focus on the interplay between active treatment and disease remission status as related to in-hospital mortality, as well as the role of disposition and goals-of-care planning for patients who are denied ICU admission after RRS calls. Further validation of our described prognostic variables, as well as further investigation into potential residual confounders, would be useful in the development of decision rules for this population.

## Conclusions

We found that hematologic oncology patients who are admitted to hospital and suffer acute deterioration experience high rates of ICU admission and in-hospital mortality. Factors associated with increased hospital mortality include ICU admission and increased number of RRS activations, while factors associated with improved survival include active malignancy treatment and longer hospital length of stay. For most of these patients, respiratory distress is the defining feature of their decompensation, and many are neutropenic at the time of RRS activation. Finally, among the subset of these patients with suspected infection, SIRS criteria are more sensitive while qSOFA criteria are more specific with respect to prognostic accuracy of in-hospital mortality.

## Additional files

Additional file 1: **Table S1.** Rapid Response System criteria at The Ottawa Hospital. (DOCX 14 kb)

Additional file 2: **Table S2.** Systemic Inflammatory Response Syndrome (SIRS) Criteria. (DOCX 13 kb)

Additional file 3: **Table S3.**Multivariable logistic regression model for prediction of mortality among Hematologic Oncology patients requiring RRS activation. (DOCX 14 kb)

Additional file 4: **Figure S1.**Patients receiving RRS activation at The Ottawa Hospital from 2012 to 2016 (DOCX 24 kb)

## Data Availability

The datasets generated and analyzed are not publicly available due to patient privacy considerations, but are available from the corresponding author on reasonable request.

## Abbreviations

ED: Emergency department
HCT: Hematopoietic cell transplant
ICU: Intensive care unit
IQR: Interquartile range
qSOFA: quick Sequential Organ Failure Assessment
RRS: Rapid response system
SD: Standard deviation
SIRS: Systemic Inflammatory Response Syndrome
