# Supplementary material for: Outcomes of hospitalized hematologic oncology patients receiving rapid response system activation for acute deterioration
Source: Crit Care. 2019 Aug 27;23:286. doi: 10.1186/s13054-019-2568-5 (PMC6712869; doi:10.1186/s13054-019-2568-5)
Supplement: Supplementary file 1 — Table S1. Rapid Response System criteria at The Ottawa Hospital. (DOCX 14 kb) [file 13054_2019_2568_MOESM1_ESM.docx]

**TableS 1:** Rapid Response System activation criteria at The Ottawa Hospital.

| **Clinical Variable** | **Description** |
| --- | --- |
| Airway | Threatened stridor; excessive secretions |
| Breathing | Respiratory rate ≤ 8 breaths/minute or ≥ 30 breaths/minute |
| Circulation | Systolic blood pressure ≤ 90 mmHg or ≥ 200 mmHg or ≥ 40 mmHg decrease  Heart rate ≤ 40 beats/minute or ≥ 130 beats/minute |
| Level of Consciousness | >2 point decrease in Glasgow Coma Scale |
| Oxygen Saturation | <90% on 50% FiO_2_ or 6 litres/minute |
| Urine Output | <100 mL over four hours |
| Other | Health care worker “worried” about the patient, needs medical assistance, failure to respond to treatment |
