# Supplementary material for: Outcomes of hospitalized hematologic oncology patients receiving rapid response system activation for acute deterioration
Source: Crit Care. 2019 Aug 27;23:286. doi: 10.1186/s13054-019-2568-5 (PMC6712869; doi:10.1186/s13054-019-2568-5)
Supplement: Supplementary file 2 — Table S2. Systemic Inflammatory Response Syndrome (SIRS) Criteria. (DOCX 13 kb) [file 13054_2019_2568_MOESM2_ESM.docx]

**Table S2:** Systemic Inflammatory Response Syndrome (SIRS) Criteria

| **Temperature** | < 36.0° C or > 38.0° C |
| --- | --- |
| **Heart Rate** | >90 beats per minute |
| **Respiratory Rate** | >20 breaths per minute |
| **White Blood Cell Count** | <4,000 per mm^3^ or >12,000 per mm^3^ |
