# Supplementary material for: Outcomes of hospitalized hematologic oncology patients receiving rapid response system activation for acute deterioration
Source: Crit Care. 2019 Aug 27;23:286. doi: 10.1186/s13054-019-2568-5 (PMC6712869; doi:10.1186/s13054-019-2568-5)
Supplement: Supplementary file 3 — Table S3.Multivariable logistic regression model for prediction of mortality among Hematologic Oncology patients requiring RRS activation. (DOCX 14 kb) [file 13054_2019_2568_MOESM3_ESM.docx]

**Table S3*:*** Multivariable logistic regression model for prediction of mortality among Hematologic Oncology patients requiring RRS activation.

| **Variables** | **Risk of Mortality** | | |
| --- | --- | --- | --- |
|  | **Point Estimate** | **95% Confidence Interval** | |
|  |  | **Lower** | **Upper** |
| **Age (every 10-year Increase)** | 1.050 | 0.884 | 1.246 |
| **Male Sex** | 0.913 | 0.570 | 1.460 |
| **Active Treatment** | 0.542 | 0.341 | 0.862 |
| **Hematologic Diagnosis** |  |  |  |
| Leukemia | 1.168 | 0.589 | 2.316 |
| Lymphoma | 1.414 | 0.751 | 2.662 |
| Myeloma | 1.594 | 0.712 | 3.567 |
| **Neutropenia** | 1.292 | 0.802 | 2.083 |
| **Elixhauser Comorbidity Score** | 1.009 | 0.974 | 1.046 |
| **Reason for RRS Activation** |  |  |  |
| Respiratory Distress | 0.909 | 0.543 | 1.521 |
| Hypotension | 1.069 | 0.931 | 1.227 |
| **Number of RRS Activations** | 2.450 | 1.628 | 3.686 |
| **ICU Admission** | 3.557 | 2.121 | 5.967 |
| **Hospital Length of Stay (every 10-day Increase)** | 0.782 | 0.702 | 0.872 |
| **Category 2 Goals-of-Care Status**^a^ | 3.570 | 1.533 | 8.315 |
| **Category 3 Goals-of-Care Status**^b^ | 2.825 | 1.423 | 5.606 |

*Area Under the Receiver Operating Characteristic (AUROC) Curve = 0.75.

^a^Category 2 allows for ICU admission and interventions (including vasoactive medications and intubation), but no cardiopulmonary resuscitation (CPR) or defibrillation. Category 3 does not allow for any CPR, defibrillation, ICU admission, or ICU interventions. Abbreviations: RRS = Rapid Response System; ICU = Intensive Care Unit.
