# Supplementary material for: Outcomes of hospitalized hematologic oncology patients receiving rapid response system activation for acute deterioration
Source: Crit Care. 2019 Aug 27;23:286. doi: 10.1186/s13054-019-2568-5 (PMC6712869; doi:10.1186/s13054-019-2568-5)
Supplement: Supplementary file 4 — Figure S1.Patients receiving RRS activation at The Ottawa Hospital from 2012 to 2016 (DOCX 24 kb) [file 13054_2019_2568_MOESM4_ESM.docx]

**FigureS 1:** Patients receiving RRS activation at The Ottawa Hospital from 2012-2016

256 Non-HCT Patients

145 HCT Patients

401 Hematologic Oncology Patients with RRS Activation

5731 Excluded (did not meet inclusion criteria)

6132 RRS Activations
